# Enhanced water splitting performance of GaN nanowires fabricated using anode aluminum oxide templates

**DOI:** 10.1039/c9ra01188a

**Published:** 2019-05-14

**Authors:** Xin Xi, Jing Li, Zhanhong Ma, Xiaodong Li, Lixia Zhao

**Affiliations:** State Key Laboratory on Integrated Optoelectronics, Institute of Semiconductors, Chinese Academy of Sciences P. R. China lxzhao@semi.ac.cn; Semiconductor Lighting Research and Development Center, Institute of Semiconductors, Chinese Academy of Sciences P. R. China; College of Materials Science and Optoelectronic Technology, University of Chinese Academy of Sciences Beijing 100049 People's Republic of China

## Abstract

Highly ordered GaN nanowires were fabricated using an anodic aluminum oxide (AAO) template. Compared to planar GaN, the GaN nanowires significantly increased the light absorption, and the saturated photocurrent increased by a factor of 5 from 0.075 to 0.38 mA cm^−2^. The photocurrent increase with the GaN nanowires is not only due to their increased surface to volume ratio and reduction in the distance for photo-generated carriers to reach the electrolyte, but also the built-in electric field, which mainly contribute to the enhancement in their water splitting ability. The GaN nanowires can lead to band bending due to their surface states and the formation of a polarized electric field to accelerate the separation of photo-generated carriers. We also established a theoretic model to simulate the band bending in the nanowires. The results showed that when the nanowire diameters are equal or bigger than the full width of depletion region, the nanowires have the maximum electric field, which improves their water splitting performance significantly. These results provide a cost-effective way for highly efficient water splitting.

## Abstract 2

Highly ordered GaN nanowires were fabricated using an anodic aluminum oxide (AAO) template.

## Graphical abstract

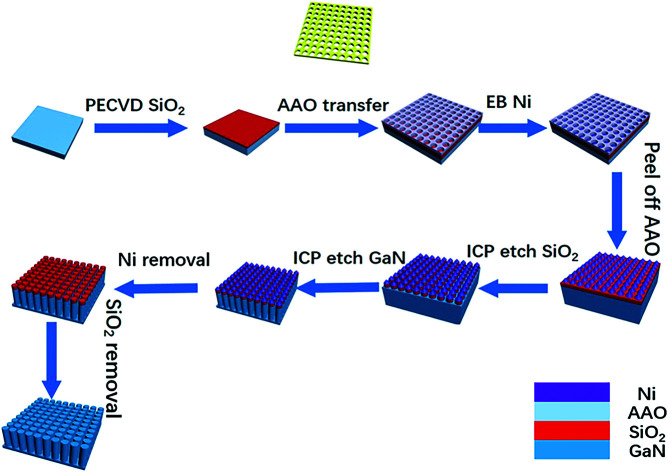

## Introduction

Water splitting has drawn extensive attention due to the generation of clean and sustainable hydrogen energy. TiO_2_ and ZnO have been widely investigated because they can be easily synthesized. However, these traditional semiconductors have low energy conversion efficiency and weak electrolyte corrosion resistance.^1,2^ Gallium nitride (GaN) has attracted considerable attention recently for water splitting due to its advantages, such as high crystalline quality, wide band gap and good material stability.^3^ The band gap of GaN is 3.4 eV at room temperature, which can split water efficiently. Besides, GaN is normally grown as a single crystal, which can transfer photo-generated carriers directly, and prevent inter-crystal scattering observed for polycrystalline materials. Furthermore, the strong ionic bond of GaN can also increase its chemical stability and prevent photo-corrosion.

GaN nanowires have strong potential applications in solar cells, nano-lasers, light emitting diodes (LED) and photoelectrochemical (PEC) water splitting.^4–6^ Compared with thin films and nano-sized powders, one-dimensional GaN nanowires exhibit a substantial enhancement in water splitting performance due to their high volume-to-surface ratio^7–10^ and reduced distance for carriers to reach the electrolyte.^11,12^ Furthermore, GaN has high chemical stability against the harsh PEC experiment conditions compared to other semiconductors.^13^ Therefore, GaN nanowires are one of the best candidates for the application of water splitting.

Traditional GaN nanowires are mainly synthesized by molecular beam epitaxy (MBE) and metal oxide chemical vapor deposition (MOCVD) using the catalyst-assisted self-organized growth or position-controlled epitaxy method.^14–16^ However, due to the turbulent gas condition in the reaction chamber and intrinsic properties of GaN, the growth of GaN nanowires is still challenging, especially to obtain a uniform morphology, homogeneous nanowires density and stable doping. The instability of GaN nanowires results in unsteady photoelectric properties in devices. In contrast, for planar GaN, its growth technology has been well developed and planar GaN has an atomic-level smooth surface with stable doping.^17–19^ Therefore, based on nano-mask technology, GaN nanowires with designed nanostructures and steady doping can be obtained *via* the top-down method using inductively coupled plasma (ICP) etching.^20,21^

Anodic aluminum oxide (AAO) membranes have been widely used for patterning (etching and evaporation masks) in various nanoscience and nanotechnology applications.^22–24^ They have a highly ordered porous structure, variable range of pore diameters and high density of pore structures. They have already been used as etching masks for the preparation of nanostructure arrays on semiconductor substrates such as Si, Ge, and GaAs.^25–27^ Highly regulated nanostructures with controlled surface-to-volume ratios can be synthesized at a lower cost than general lithographic techniques.^28–30^

Although significant work on AAO templates in pore array application has been carried out, the fabrication of GaN nanowires has rarely been reported. Herein, highly ordered GaN nanowires with uniform diameters were prepared using the AAO templates for the first time. Due to their increased surface state, large surface-to-volume ratio, decreased distance between the carriers to the electrolyte and surface state, the GaN nanowires exhibited excellent water splitting properties. Their photocurrents reached 0.38 mA cm^−2^, which is ∼5 times that of planar GaN under 90 mW cm^−2^ Xe lamp illumination. Thus, our work demonstrates a new method for developing GaN nanowires as photo anodes for highly efficient water splitting application.

## Experimental

A 2 μm n-doped GaN layer was firstly grown on a sapphire wafer using MOCVD. Trimethyl gallium gas was used as the Ga source and ammonia gas was used as the N source. During the growth, hydrogen was used as the carrier gas. The carrier concentration of the grown GaN was 3 × 10^18^ cm^−3^ and the mobility was about 320 cm^2^ V^−1^, as determined by Hall effect measurements.

**Fig. 1:**
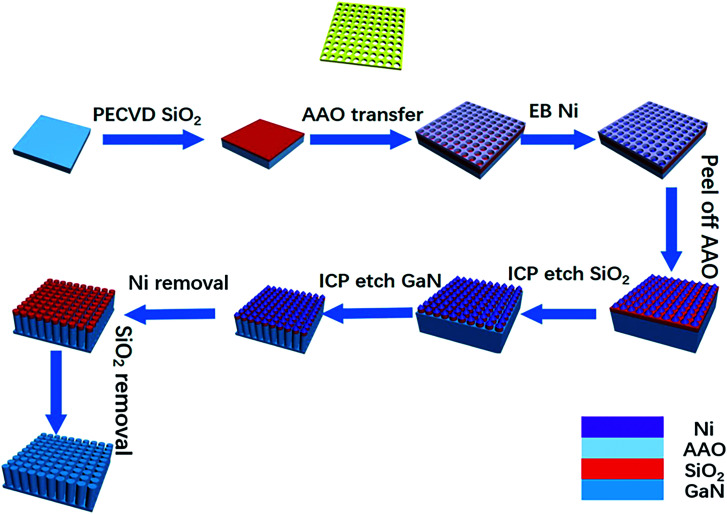
Illustration of the fabrication of GaN nanowires using an AAO mask as the pattern template.

Fig. 1 illustrates the fabrication of the GaN nanowires using AAO as a mask. First, 200 nm SiO_2_ was grown on a GaN substrate *via* plasma enhanced chemical vapor deposition (PECVD).^31^ Then, three types of AAO membranes with a pore size of 60, 100 and 300 nm were transferred onto the SiO_2_. The diameters and pitch of holes for AAO were 60 and 100, and 100 and 140, 300 and 450 nm, respectively. A 30 nm thick Ni mask was deposited on the SiO_2_*via* electron beam evaporation (EB). After dipping the patterned GaN into water to remove the AAO above the substrate, a remnant Ni particle array was left on the SiO_2_. Using the Ni arrays as the etching mask, the pattern was transferred to SiO_2_*via* inductively coupled plasma (ICP) etching. By continuously etching the underlying GaN film, GaN nanowires were formed through the pattern transfer. The target length of the GaN nanowires under the different masks was ∼1 μm. Finally, the residual SiO_2_ and Ni were removed by dipping in hydrofluoric acid and nitric acid to achieve GaN nanowires.

The morphology of the GaN nanowires was determined using scanning electron microscopy (SEM, Hitachi S-4800). The crystal structure of the nanostructures was characterized using a Bruker D8 X-ray diffractometer (XRD) equipped with Cu Kα line (*λ* = 0.15419 nm) radiation. Raman measurements were performed using a Horiba-Jobin Yvon LabRAM ARAMIS system with 420 nm radiation from a 20 mW HeNe laser. The crystal properties of the nanowires were characterized by photoluminescence (PL) at room temperature using a laser (325 nm) as the excitation light source using the Horiba-Jobin Yvon LabRAM ARMIS system. The optical properties of the different GaN nanowires were examined using a UV-VIS-NIR spectrophotometer (Varian Cary-5000). Photoelectrochemical measurements (PEC) on the GaN nanowires were carried out under an Xe arc lamp (300 W). The light intensity was adjusted about 90 mW cm^−2^. A CHI 660E electrochemical workstation was used to control and adjust the applied voltage and current. A three-electrode quartz cell was used to carry out the water splitting experiment. The fabricated GaN nanowires were used as the photo anode and a Pt plate was used as the counter electrode. The electrolyte was 1 M NaOH solution. The *I*–*V* curves were measured by adjusting the voltage through the electrochemical workstation with the light on and off. The *I*–*t* curves were measured at 0 V with and without light.

## Results and discussion

**Fig. 2:**
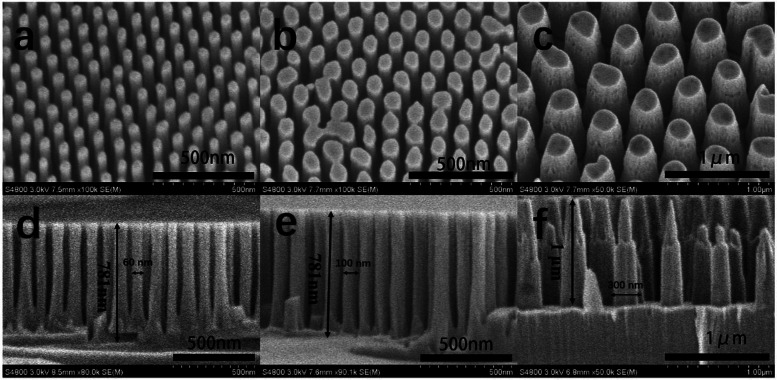
Top tilted SEM images (tilted at 30° angle) (a)–(c) and sectional SEM images (d)–(f) of the GaN nanowires with diameters of 60, 100 and 300 nm, respectively.

The top-view scanning electron microscopy (SEM) images of the GaN nanowires are shown in Fig. 2(a)–(c). The diameters of the GaN nanowires are 60, 100 and 300 nm, respectively, which are consistent with the AAO surface pattern. This suggests that the pattern of AAO was successfully transferred to the GaN nanowires with little side etching effect. The sectional SEM images of the GaN nanowires are shown in Fig. 2(d)–(f), from which the heights of the nanowires were determined to be ∼0.78, 0.78 and 1.00 μm, respectively. Also, their sidewalls were observed to be relatively smooth.

Compared to planar GaN, the surface area of the side walls of the GaN nanowires mainly contribute to their increased surface area. Therefore, for each type of GaN nanowire, the increased surface area can be calculated using eqn (1).

1 *S* = 2π*Rh*

where, *S* is the increased special area surface per square micron, *R* is the radius of the GaN nanowires, and *h* is the height of the GaN nanowires. The increased surface area per square micron for the nanowires with diameters 60, 100 and 300 nm is 29.4, 34.1 and 9.3 μm^2^, respectively, compared to planar GaN. The additional surface-to-volume area can enhance the water splitting performance by increasing the light absorption and chemical reaction sites.

**Fig. 3:**
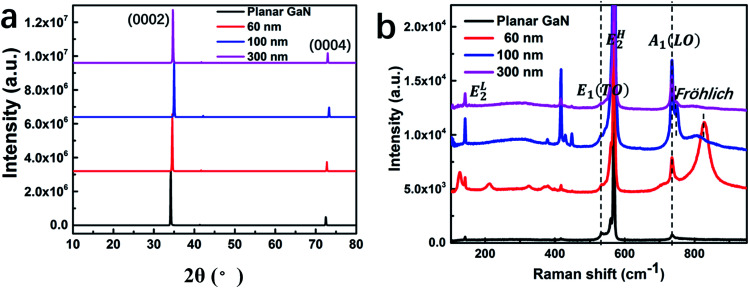
XRD (a) and Raman (b) spectra for the GaN nanowires with diameters of 60, 100 and 300 nm and corresponding planar GaN.

Fig. 3(a) shows the XRD spectra of the different GaN nanowires and corresponding planar GaN. Only two diffraction peaks of (0002) and (0004) can be observed,^32^ which suggests that GaN grew along the (0001) facet with good single crystal quality.^8^ The (0002) facet for the GaN nanowires with diameters of 60, 100, 300 and corresponding planar GaN was observed a 2*θ* values of 34.70°, 35.01°, 34.53° and 34.19°, respectively. Due to the lattice mismatch between the sapphire substrate and GaN, tensile strain existed during the growth of GaN, which is normal. The nanowire structure can help to relax the tensile strain effectively, which results in a decrease in the lattice parameters. Therefore, the XRD peaks shifted to a slightly higher angle compared to that of planar GaN. Meanwhile, the surface areas of the GaN nanowires were 29.4, 34.1 and 9.3 μm^2^ per square micron for the GaN nanowires with a diameter of 60, 100 and 300 nm, which correspond to the XRD peaks of 34.70°, 35.01°and 34.53°, respectively, indicating that a larger surface area can lead to more effective strain relaxation more.

Fig. 3(b) shows the Raman spectra of the different GaN nanowires and planar GaN. GaN crystallizes in the hexagonal wurtzite structure, which belongs to the space group *C*^4^_6v_-*P*6_3_*mc*.^33^ The peaks at 143, 556, 568 and 733 cm^−1^ are attributed to the first-order phonons of E^L^_2_, E_1_(TO), E^H^_2_ and A_1_(LO), respectively.^34^ The peaks at 315 and 418 cm^−1^ are related to an acoustic phonon overtone.^35^ With a decrease in the diameter of the GaN nanowires, a peak at 752 cm^−1^ appeared for GaN the nanowires with a diameter of 100 nm.^36^ The slight red-shift of the A_1_(LO) mode is attributed to phonon confinement within the nano-dimensional system.^37^ Furthermore, the peak intensity of E^L^_2_, E_1_(TO), E^H^_2_ and A_1_ (LO) for the GaN nanowires increased significantly compared to that of planar GaN. This is due to the increased special surface area, which results in more molecular vibration when the GaN nanowires are exposed to an incident laser.^38^ Meanwhile, a new Raman mode centered at about 815 cm^−1^ was observed for the GaN nanowires with a diameter of 60 nm. This mode is the surface mode (Fröhlich mode) of GaN nanocrystals, as reported for a porous GaN film.^39^

**Fig. 4:**
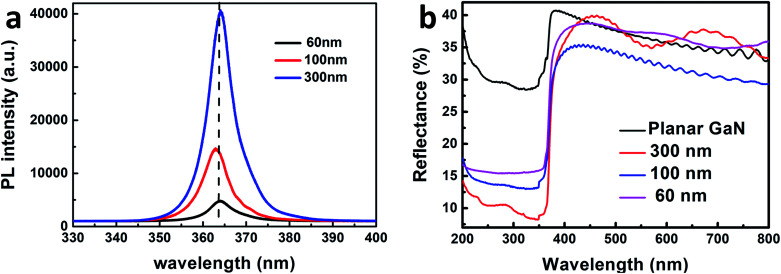
Room temperature PL (a) and reflectance spectra (b) of the GaN nanowires with diameters of 60, 100, 300 nm and planar GaN.

To investigate the light absorption of the GaN nanowires, their PL spectra and reflectance spectra were measured. The room temperature PL spectra of the GaN nanowires are shown in Fig. 4(a). The PL peak for the GaN nanowires is located at 364 nm, which corresponds to the bandgap of GaN. As the diameter of the GaN nanowires increases, the PL peak intensity increased obviously. This is due to the increased depletion region for the separation of carriers, which will be discussed later. The PL peak of the GaN nanowires blue-shifted by 100 nm, which is mainly due to the relaxation of tensile strain. This is because the surface area can eliminate tensile strain effectively^14^ and the surface area of GaN with a diameter of 100 nm is larger than that for the GaN nanowires with a diameter of 60 nm. Therefore, GaN nanowires with 100 nm will have more strain relaxation and blueshifts than that of the GaN nanowires with 60 nm, which is also consistent with the XRD results.^40^

The diameters and pitch of holes for AAO are 60 and 100, and 100 and 140, 300 and 450 nm, respectively. During the ICP etching process, more GaN was etched for the diameter of 60 nm, which resulted in the most surface defects among all the GaN nanowires. The surface defects will generate defect energy, which traps the photo-generated carriers.^11^ Thus, less photo-generated electron–hole pairs are recombined during the PL measurement, which results in a lower PL intensity.

To understand the light absorption properties of the GaN nanowires, their diffusive reflectance spectra were measured, as shown in Fig. 4(b). The diffusive reflectivities for the planar GaN and different nanowires are 28%, 17%, 13% and 6% in the GaN absorption spectrum, respectively. Compared to planar GaN, the GaN nanowires exhibited an extremely lower reflectance, which indicates that the GaN nanowires have stronger light absorption due to their large surface-to-volume ratio. In addition, the reflectance peak red-shifted by ∼45 nm, which is mainly due to the strain relaxation.^41^

**Fig. 5:**
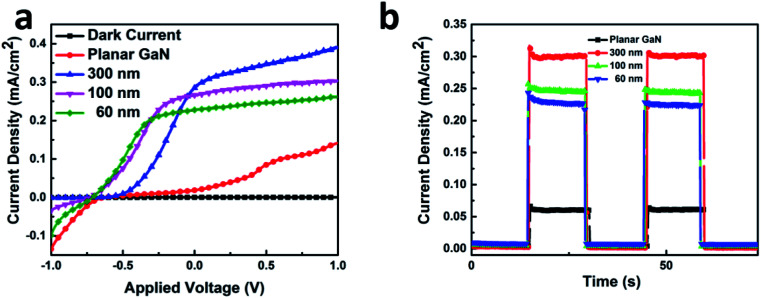
Linear sweep voltammetry (a) and time dependent photocurrent density (b) of GaN nanowires with diameters of 60, 100, and 300 nm and planar GaN.

The PEC performances were examined in a three-electrode electrolytic cell using the GaN samples as the working electrode. Fig. 5(a) shows the linear sweep voltammetry properties. The black line represents the dark currents for all the samples, which is significantly low at 0.002 mA cm^−2^. At ∼0.5 V, the photocurrent of the planar GaN was saturated at ∼0.07 mA cm^−2^. In contrast, for the GaN nanowires with diameters 60, 100 and 300 nm, the saturated photocurrents were ∼0.25, 0.29 and 0.38 mA cm^−2^, respectively. This suggests that more photo-generated carriers were generated under illumination for the GaN nanowires, which can convert solar energy to photocurrent more efficiently. The quantum efficiency can be estimated using eqn (2):2
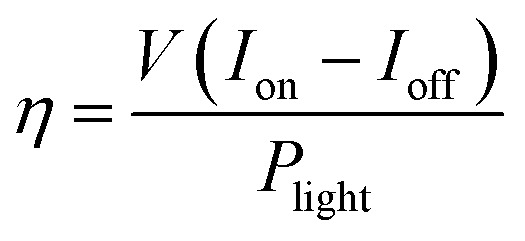


The quantum efficiencies were calculated under 1 V bias, which are 0.39 × 10^−2^, 0.30 × 10^−2^, 0.26 × 10^−2^ and 0.14 × 10^−2^, respectively, for the GaN nanowires with diameters of 60, 100, and 300 and planar GaN.

The larger surface-to-volume ratio of the GaN nanowires can increase the contact interface between the semiconductor and electrolyte. A greater contact area will induce more reaction sites at the surface of GaN and facilitate the water splitting reaction. Meanwhile, the surface state will result in band bending at the surface.^9^ A depletion region between the surface and inner bulk GaN will be formed due to the difference in Fermi level. This depletion region will generate a built-in electric field, which separates the photo-generated carriers more promptly. The rapid transportation of holes to the surface can photo-catalyze water directly to oxygen and reduce the recombination of holes in the transportation process. Therefore, the GaN nanowires exhibited an enhanced water splitting performance compared to that of the planar GaN.

In addition, the GaN nanowires can reduce the distance for carriers to reach the surface because the radial and lateral transportation of GaN nanowires is more efficient than the only perpendicular transportation for planar GaN. This can prevent the recombination of carriers during long-distance transport and make photo-generated carriers react with water more efficiently.^42^

The water splitting experiments were carried out under zero-bias. Fig. 5(b) shows the time-resolved current curve. The GaN nanowires with a diameter of 300 nm exhibited the highest photocurrent of ∼0.30 mA cm^−2^. The photocurrent of the planar GaN was about 0.06 mA cm^−2^, which is only one fifth of that of the GaN nanowires with a diameter of 300 nm. For the GaN nanowires with the diameters of 60, 100 and 300 nm, their photocurrents were 0.22, 0.25 and 0.30 mA cm^−2^, respectively. All the GaN nanowires exhibited a significant enhancement in photocurrent compared with the planar GaN. Also, the photocurrents became larger as their diameter increased.

As previously calculated, the increased surface area per square micron for the GaN nanowires with diameters 60, 100 and 300 nm was 29.4, 34.1 and 9.3 μm^2^, respectively. If the main factor contributing to the enhancement of photocurrent is specific surface area, the photocurrent for the GaN nanowires with a diameter of 100 should be the largest. However, this was not the case.

**Fig. 6:**
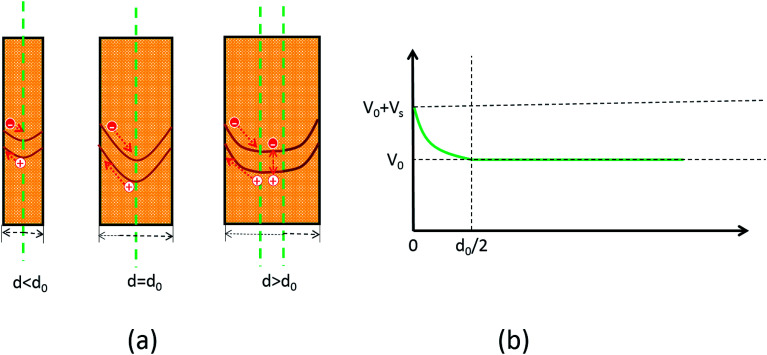
Schematic diagram of the Fermi level at the GaN nanowire surface.

There are many factors influencing the water splitting property, such as surface band bending, surface-to-volume ratio and distance between the carriers and electrolyte, and the dominant factor for water splitting property is still related to the surface band bending.^41^ To understand the water-splitting properties and investigate the band bending of the GaN nanowires, we qualitatively analyzed their diameters and band bending. According to Poisson eqn (3):3
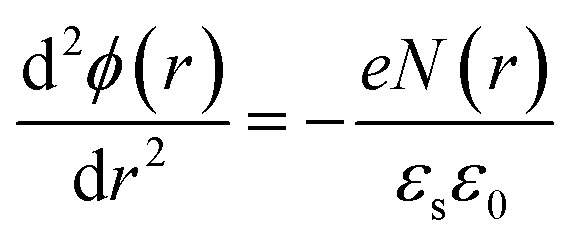
where, *ϕ*(*r*) represents the surface potential, *e* and *N*(*r*) are elementary charge and the carrier density at position *r*, and *ε* and *ε*_0_ are electric constants for GaN and vacuum permittivity, respectively. Due to the difference in the Fermi level between the surface state and GaN, electrons flow to the surface, which results in the band bending upwards to the surface. As shown in Fig. 6(a), if the diameters of the nanowires are smaller than the complete depletion region, the bands cannot bend enough to get the maximum electric field, and if the diameters are equivalent or bigger than the whole width of the depletion region, the most curved band bending can generate the maximum electric field to separate the carriers effectively. Therefore, the minimum diameter for the fast separation of carriers is twice the width of the depletion region. The width of the depletion region can be calculated by the boundary condition, as shown in Fig. 6(b).4
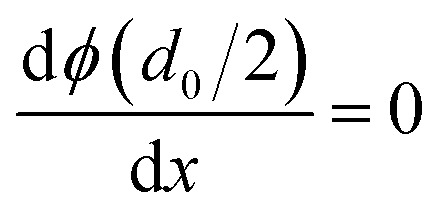

5 *ϕ*(0) = 0

6 *ϕ*(*d*_0_/2) = *V*_s_

7
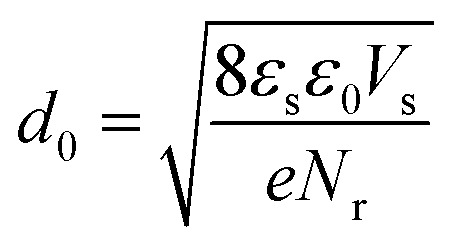
where, *V*_s_ is the difference between the surface state and GaN.

The width of the depletion region was calculated to be 70 nm; thus, the diameters of the GaN nanowires should be larger than 140 nm. When the diameters were thinner than 140 nm, the band bending was not enough to reach the maximum electric field. Therefore, the photocurrent of the GaN nanowires became larger with an increase in diameter when the diameter is smaller than *d*_0_ 140 nm. However, when the diameters are equal to or bigger than the width of depletion region, the nanowires will have the maximum electric field, which will improve their water splitting performance significantly.

## Conclusion

In conclusion, GaN nanowires were fabricated using AAO as a pattern template. The GaN nanowires exhibited an ∼5 times enhancement in water splitting ability compared to planar GaN due to their increased surface-to-volume ratio. The nanowires not only result in more reaction sites between the electrolyte and photo anode, but also reduce the transportation distance of photo-generated carriers to the solution. In addition, the more surface states of the GaN nanowires result in an inner electric field, which can separate the photo-generated carriers more effectively. We qualitatively analyzed the diameters and band bending, and found that the best diameter of the GaN nanowires is ∼140 nm. When the diameter is equivalent to or bigger than 140 nm, the nanowires will have the maximum electric field, which will improve their water splitting performance.

## Conflicts of interest

There are no conflicts to declare.

## Supplementary Material
